# A Device to Predict Short-Term Healing Outcome of Chronic Wounds

**DOI:** 10.1089/wound.2019.1064

**Published:** 2020-04-08

**Authors:** Hong Vu, Ashwin Nair, Lan Tran, Suvra Pal, Jon Senkowsky, Wenjing Hu, Liping Tang

**Affiliations:** ^1^ Progenitec, Inc., Arlington, Texas.; ^2^ Department of Mathematics, University of Texas at Arlington, Arlington, Texas.; ^3^ Texas Health Physician's Group, Arlington, Texas.; ^4^ Department of Bioengineering, University of Texas at Arlington, Arlington, Texas.

**Keywords:** chronic wound, wound pH, wound dressing, clinical study, healing rate

## Abstract

**Objective:** While myriads of studies have suggested that a survey of wound pH environment could indicate wound healing activities, it is not clear whether wound alkalinity can be used as a prognostic indicator of nonhealing wounds. Currently available systems cannot reliably assess the pH environment across wounds, which is the objective of this study.

**Approach:** A disposable device, DETEC^®^ pH, was developed and characterized on its ability to map wound alkalinity by pressing a freshly recovered wound dressing against its test surface. By comparing the wound's alkalinity and size reduction rates (∼7 days) following pH measurement, we assessed the capability of wound alkalinity to prognosticate subsequent short-term wound size reduction rates.

**Results:** The device had high accuracy and specificity in determining the alkalinity of simulated wound fluids soaked onto wound dressing. The type of wound dressing type had an insignificant effect on its detection sensitivity. Upon testing discarded wound dressings from human patients, the device quickly determined alkaline and acidic wounds. Finally, statistical analyses of wound size reduction rates in wounds with various alkalinities confirmed that wound alkalinity has a strong influence on, at least, short-term wound healing activity.

**Innovation:** Without directly contacting the patient, this device provides a quick assessment of wound alkalinity to prognosticate immediate and short-term wound healing activities.

**Conclusion:** DETEC^®^ pH may serve as a prognosis device for wound care specialists during routine wound assessment to predict wound healing progress. This information can assist the decision-making process in a clinical setting and augur well for chronic wound treatment. DETEC^®^ pH can also be used as an aid for home health care nurses or health care providers to screen nonhealing wounds outside clinics.

**Figure:**
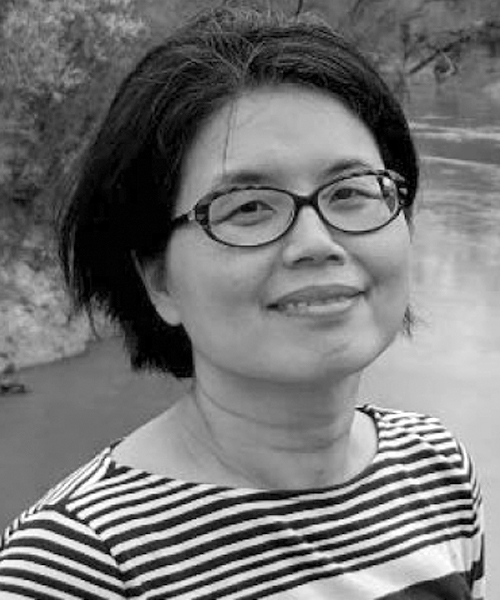
**Wenjing Hu, PhD**

**Figure:**
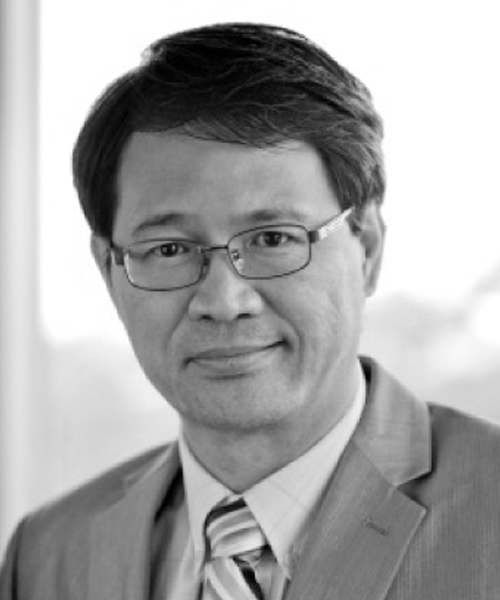
**Liping Tang, PhD**

## Introduction

Chronic nonhealing wounds cost more than U.S. $25 billion every year.^1^ Preliminary assessment of these wounds is conducted through visual examination,^2^ while additional assessments are done using wound photography, digital tracing, and microbiological culture.^3,4^ Unfortunately, these methods cannot predict the progress of healing. Pulse oximetry can measure oxygen saturation and detect poorly healing areas,^5,6^ but its output may be influenced by patients' movement and can be unreliable.^7^ Several imaging techniques, such as ultrasound imaging, magnetic resonance imaging, laser Doppler perfusion imaging, are also used to confirm ischemic or venous etiology for the wound and the presence of infection or ischemia.^8^ Although the assessments of biochemicals, including procollagen, elastin, and hyaluronic acid, in wound exudates have been evaluated on their potential to determine healing activities,^9^ these biochemical assessments are limited by the wound exudate availability, tedious procedure, and high cost. Due to these limitations, there is no established method for prognosticating wound healing activities at the bed side.^10^ Since real-time wound prognosis can greatly improve wound treatment decision with optimal healing outcome, there is an urgent need for a prognostic tool that can quickly assess chronic wound healing activities.

Surface pH is a useful biophysical parameter that can be used to measure the wound environment and evaluate healing activities.^11^ Chronic wounds typically have an alkaline environment,^12–15^ with lower healing rates compared with wounds with pH closer to neutral or acidic.^16–18^ For example, alkaline wounds have impaired synthesis of extracellular matrix molecules leading to a cessation of healing responses and a more chronic wound environment.^12,19^ Generally, prevalence of alkalinity is associated with slow healing. Since inflammatory cell products are mostly acidic, alkaline wounds are associated with weak immune responses. Due to the weak immune responses, alkaline wounds provide an excellent environment for bacterial colonization, which may lead to wound infection.^20,21^ On the other hand, it has been found that wound healing processes are accompanied with an acidic environment.^17,19,22^ Furthermore, many studies have shown that prolonging the acidification of the wound environment accelerates healing by significantly inhibiting the growth of infection causing microorganisms and creating a hypoxic environment that is favorable for healing.^23–25^ An earlier study on similarly sized ulcers in around 36 patients found a highly significant improvement in the healing rate (sq. mm/day) in patients with prolonged acidification of the ulcer as compared with controls.^26^ In fact, Manuka honey dressings are used in chronic wound treatments due to its ability to maintain an acidic environment.^27^ Nonhealing chronic wounds that had no reduction in wound size over the 3 weeks preceding treatment, exhibited a significant reduction (almost 30%) in wound size when treated with these dressings in just 2 weeks.^14^ These results support that wound alkalinity may be used as an indicator for the prognosis of wound healing status.

A number of publications have voiced support for detecting pH to identify alkalinity and acidity as valuable wound biomarkers, either as a standalone or supplementary tool.^17,28–33^ It must be noted that most chronic wounds do not have a uniform environment and have various extents of healing activities at different regions. Hence, assessment of the entire wound landscape has been emphasized.^34^ Standard pH meters can be used to assess the pH, but have to be kept in prolonged contact with the wound. Also, they provide an average pH only at the single point of contact instead of the entire wound.^35–37^ pH strips have been widely used in detecting infections in the wound as well as in the urinary tract, vagina, and in the evaluation of salivary pH in human immunodeficiency virus-positive individuals. However, like the pH meter, pH strips cannot be used to assess the entire wound landscape. While several wound fluid collecting devices and methods have been proposed in patents and publications,^38–40^ such products are not commercially available. Also, as collected and mixed wound fluids will not reflect the pH of specific regions of the wound, they may not best reflect the healing activity across the wounds. Therefore, there is a need for a device that can rapidly assess the alkalinity of the entire wound area.

**Figure 1.**
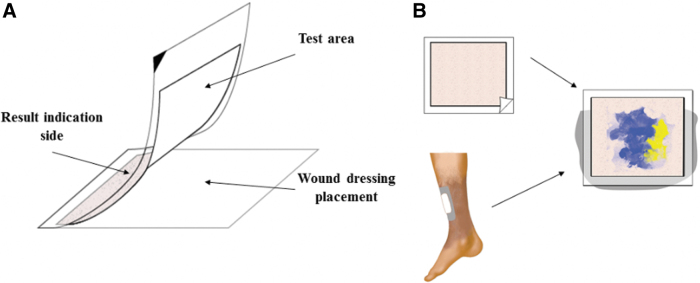
**(A)** Overview of DETEC^®^ pH. **(B)** DETEC^®^ pH testing scheme. Existing dressing is removed from the wound and placed on the wound dressing holder on the device. Within 60 s, wound imprint from the dressing appears on the film, with the color map reflecting the pH pattern of wound environment.

In the current study, we have developed a multilayered wound alkalinity monitoring system, DETEC^®^ pH (Fig. 1A), which can help clinicians assess wound environment by testing discarded wound dressings, thereby avoiding contact with the wound bed directly, as shown in Figure 1B. Using simulated wound fluids (SWF) with different pH, we evaluated the ability of the device to indicate acidity and alkalinity on clinically used wound dressings through *in vitro* studies. In addition, by testing various wound dressings from different manufacturers with this device, the influence of wound dressing materials on determination of alkaline/acidic environment was also assessed. Subsequently, the capability of DETEC^®^ pH to assess alkalinity of the wound environment was investigated using discarded wound dressings isolated from human patients with chronic wounds. Finally, using statistical analyses, the relationship between wound alkalinity and postmeasurement wound healing rates was determined to support the usefulness of measuring wound alkalinity as a prognostic indicator of wound healing activities in chronic wounds.

## Clinical Problem Addressed

The clinical standard of care in wound healing assessment is speculative and relies on the experience of the clinician. While wound alkalinity has been highlighted in many studies as an important predictor of wound healing status, commercial products, such as pH skin probe and pH strips, cannot accurately and easily assess the alkalinity across wounds. In addition, it is not clear whether it can be used to prognosticate short-term wound healing rate immediately following the measurement. This article describes the development of an inexpensive portable device to rapidly distinguish alkaline and acidic wounds. The results may be used to predict the short-term healing progress of chronic wounds and to screen the nonhealing wounds.

## Materials and Methods

### Materials

Grade 4, Whatman™ Filter membranes with a thickness of 0.14 mm and 20–35 μm pore size were manufactured by GE Healthcare. Transparent vinyl films and opaque light-colored vinyl films were purchased from Sanshui Xinli Paper Mucilage Glue Co., Ltd (Foshan, China). Nitrazine Yellow, Bovine Serum Albumin, Calcium Chloride, Sodium Chloride, and Tris Methylamine were purchased from Sigma-Aldrich (St. Louis, MO).

Twenty common wound dressings were chosen for this study are listed below:

Curity™ gauze sponges (Covidien/Medtronic, Minneapolis, MN), Cutimed^®^ Siltec Sorbact^®^ (BSN medical, Inc., Charlotte, NC), Mepilex^®^ Border (Mölnlycke Health Care US, LLC., Norcross, GA), Enluxtra (OSNovative Systems, Inc., Santa Clara, CA), Hydrofera Blue^®^ (Hollister Incorporated, Libertyville, IL), Aquacel^®^, DuoDerm^®^ CGF™, DuoDerm Hydrocolloid (ConvaTec, Inc., Oklahoma City, OK), Mesalt^®^ (Mölnlycke Health Care US, LLC.), Mepitel^®^ One (Mölnlycke Health Care US, LLC.), Xeroform^®^ Occlusive Petrolatum Gauze Strip (Covidien/Medtronic), Medihoney^®^ (Derma Sciences, Inc., Plainsboro, NJ), Aquacel Ag (ConvaTec, Inc.), Mepitel Ag (Mölnlycke Health Care US, LLC.), Restore Contact Layer with Silver (Hollister Incorporated), InterDry^®^ Ag (Coloplast, Minneapolis, MN), Iodoflex* Cadexomer Iodine (Smith & Nephew, Fort Worth, TX), PROMOGRAN PRISMA™ Matrix (Systagenix, San Antonio, TX). Knitted fabric dressings—Curity Non-Adherent Strips (Covidien/Medtronic).

### Methods

#### Fabrication and utilization of pH membrane

A pH indicator, 98–100% Nitrazine Yellow powder, was coated on one side of cellulose-based filter membranes with a coverage of ∼10 mg/cm^2^, by modifying an earlier process.^41^ Subsequently, the membrane was laminated with a cold roll laminator using a transparent film with a transparent indicator side. The wound gauze/dressing contacting side also referred to as the test area is then sealed by attaching a sealable plastic film, which is used as a holder for the discarded dressing/gauze (Fig. 1A). The device had a dimension of 10 cm × 10 cm to accommodate most wound dressings. The design of the device also facilitates placing multiple devices end to end in case of larger dressings. Upon pressing the wound dressing against the test area, a visible color map (reversed around a vertical axis) begins to appear on the indicator side that indicates alkalinity (either alkaline or acidic) of wound fluid soaked on the dressing (Fig. 1B).

#### In vitro testing of DETEC^®^ pH performance

##### Influence of various pH on DETEC^®^ pH output

SWF was prepared as documented earlier.^42–44^ The pH was adjusted to prepare SWF with various pH (3 to 9). Around 5 μL of this SWF was dropped on the test pad of the device and the color change observed. SWF with various pH was also dropped on a wound dressing that was then tested using DETEC^®^ pH to observe development of a pH map on the device that reflected the pH on the dressing.

##### Influence of wound dressing on wound fluid pH

SWF with different pH (pH 4, 6, and 8) was aliquoted into 30 wells on 12-well plates. Similar to earlier studies, wound dressings (10 mg/mL of SWF),^45^ were immersed into 5 of these wells per pH (total 15 wells with wound dressings) that were sealed and stored at room temperature (24°C to 28°C) for 3 h and days 1, 3, 5, and 7. At these specific time points, pH of SWF alone and on wound dressing were then measured using pH surface probe (HI 99181; HANNA Instruments, Smithfield, RI) as described earlier.^45,46^

##### Influence of wound dressing on DETEC^®^ pH measurement

Various wound dressings were soaked with SWF with a ratio of 1% w/v in six-well plates and incubated at room temperature for up to 24 h.^47^ The pH of SWF in solution and on dressings was then measured using DETEC^®^ pH.

**Figure 2.**
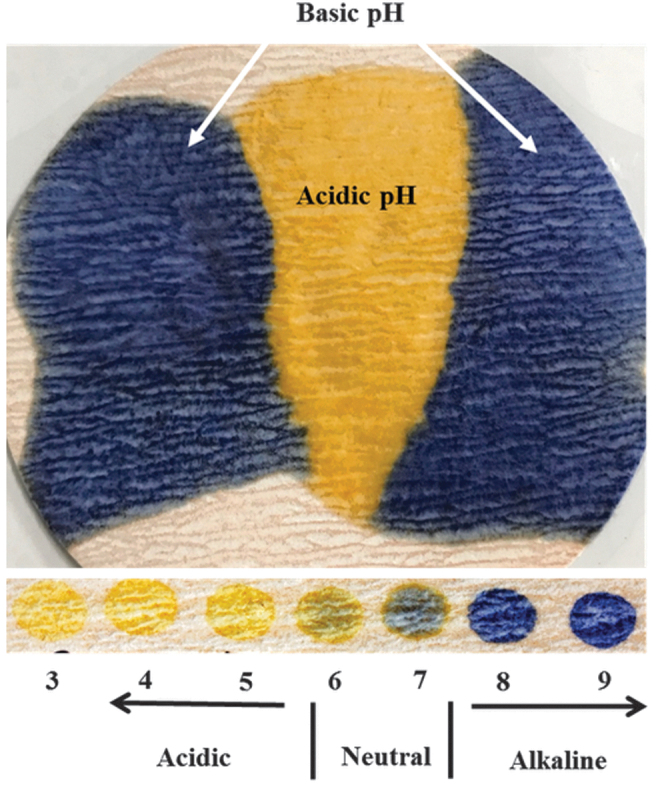
DETEC^®^ pH color change after contacting a wound gauze soaked in SWF with various pH (*upper panel*). Change in color in response to various pH (*lower panel*). SWF, simulated wound fluid.

Additionally, each dressing was cut into 1 × 1 cm area with single layer. SWF with a pH of 7 was dropped on prepared dressing with various volumes, which was then tested using DETEC^®^ pH. The color change was observed and matched with expected color that corresponds to pH of SWF. SWF with a pH of 7 was chosen as it is approximately at the midpoint of the device's color scale (Fig. 2) and hence pH shift in either direction would be easy to observe as a color change toward blue or yellow.

#### Clinical tests on DETEC^®^ pH

A single-blind study was conducted at the wound care clinic at Texas Health Arlington Memorial Hospital on 51 subjects with 121 measurements in accordance with the principles of Helsinki Declaration. The testing personnel from Progenitec did not receive any information related to the specific treatment that was being administered. Patients were chosen by wound care staff based on the following inclusion criteria: adult male or female 18 years of age or older; has diabetic ulcers, pressure ulcers, venous stasis; has a nonhealing and/chronic wound for at least 30 days; and subject's wounds were measured using standard linear measurement methods and area must be at least 1.5 cm^2^. Our patient group, even though diverse, suffered from similar underlying disease conditions—diabetes and venous stasis. Standard protocols in wound care were followed in the clinic as detailed in earlier publications.^48–50^ No special procedure, for example, skin graft or surgical debridement, was done on the wound during this study. The demographic information for all patients can be seen in Table 1. Subjects visited wound clinic and were given professional wound care every 1 to 2 weeks. All subjects visited the clinic at least twice, and each visit was between 4 and 15 days apart.

**Table 1.** Demographic characteristics of patients with chronic wounds

	Group	n (%)
Total	Patient numbers	51
	Median age (years)	64
	Gender (female)	20 (39)
Ethic	Non-Hispanic white	32 (63)
	African American	8 (16)
	Mexican American	10 (20)
Alcohol use	Formerly	4 (8)
	Currently	6 (12)
Tobacco use	Formerly	14 (27)
	Currently	15 (29)
Health problems	Type 2 diabetes	34 (67)
	Hypertension	25 (49)
	High cholesterol	14 (27)
	High blood pressure	9 (18)
	Chronic obstructive pulmonary disease	12(24)
	Anemia	2 (4)
	Hypothyroid	2 (4)
	Hepatitis	3 (6)
	Coronary artery disease	6 (12)
	Congestive heart failure	5 (10)
	Kidney disease	10 (20)
Wound size range (cm^2^)		1.95 to 172.5
Wound size average (cm^2^)		33.48
Wound location	Leg	14 (27)
	Foot	18 (35)
	Ankle	3 (6)
	Heel/Achilles	5 (10)
	Thigh	2 (4)
	Sacrum/hip	6 (12)
	Axillae	2 (4)
	Below-the-knee amputation	1 (2)

During each visit, old dressing on a subject's wound was removed and tested immediately by the wound care specialist. Photographs of the pH map on DETEC^®^ pH were captured. Color pattern on the DETEC^®^ pH film was observed by wound care specialist and categorized using the following categories: Alkaline, if the film only turned blue; Acidic, if yellow areas were seen on the film. In addition, wound size was measured during each visit by wound care specialists. We statistically analyzed the influence of wound categories on subsequent wound size reduction rate. Wound size reduction rate was calculated as follows:

$$\left[\left(\frac{wound\;size\;visit\;2-wound\;size\;visit\;1}{wound\;size\;visit\;1}\right)÷\left(number\;of\;days\;between\;visits\right)\right]\times 100$$

### Statistical analysis

A two-sample nonparametric Wilcoxon rank sum test was performed to compare the wound healing rates in both alkaline and acidic wounds at a significance level of 5% or 10%.

## Results

### Performance characteristics of DETEC^®^ pH

The ability of DETEC^®^ pH to distinguish wound fluid alkalinity was determined using SWF at different pH. As seen in Figure 2, the device turns a yellow color after exposure to acidic SWF with pH <6. At pH of 6 to 7, the device has a light green color. When exposed to alkaline SWF with pH >7.5, the device shows a dark blue color. These results concur with the general shift in colors for the indicator dye, Nitrazine Yellow, in response to change in pH. Upon contact with a wound dressing soaked in SWF with various pH, a distinct pattern of colors appeared that correlated with the alkaline pH on the sides of the dressing with an acidic center region (Fig. 2). This supports that the device can not only respond to pH but also show different pH on the same wound dressing.

### Effect of wound dressings on the pH of simulated wound fluids

**Figure 3.**
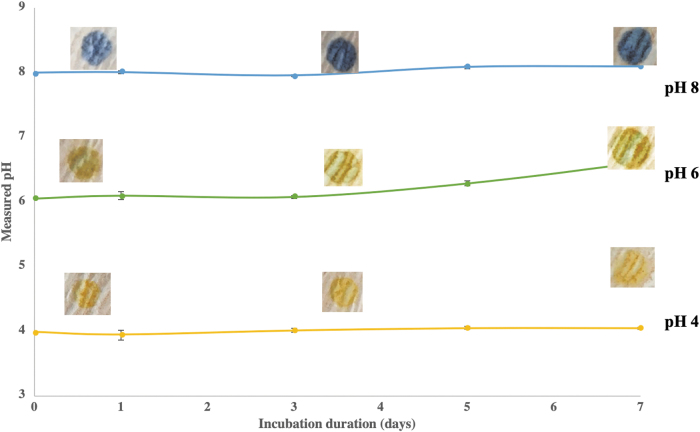
Change in pH of SWF during 7 days incubation with a wound dressing (Curity Sponges).

The device evaluates wound pH environment by measuring pH of wound exudate adsorbed on to wound dressings. To test the feasibility of this concept, we evaluated the influence of wound dressing materials on wound exudates' pH. First, we measured the pH changes in different SWFs incubated with Curity wound dressing for different periods of time (up to 7 days). The study was done over 7 days to reflect how often dressing is changed (typically every 4 to 7 days) in chronic wound patients to show the suitability of using SWF for subsequent *in vitro* tests. We found no discernible changes between the pH of the wound exudate in solution and upon contacting a dressing at any given time point (Fig. 3). In agreement with a previous publication, our findings show that wound dressings only have a transient and minor effect on pH (<0.5 pH) that usually diminishes after 24 h.^46^ It should be noted that such minor changes have no apparent influence on the outcome of our device measurement because the intended use of this device is to distinguish between alkaline (totally blue) and acidic (mainly or partially yellow or green) wounds. The qualitative output of the device did not change in any of the tested wound dressings. For example, in case of PRISMA or any of the other wound dressings where changes in pH values were observed, pH of 8 was indicated correctly as alkaline.

**Figure 4.**
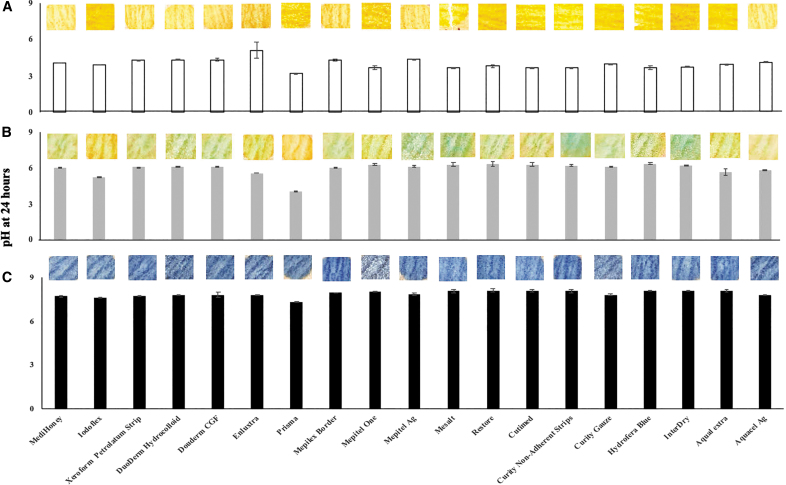
Effect of wound dressing on SWF with **(A)** pH 4, **(B)** pH 6, and **(C)** pH 8. Images of DETEC^®^ pH output for each dressing at a specific pH is shown.

There are a wide variety of commercially available wound dressings designed for treating different wound conditions. The potential influence of various commercially available wound dressings on the pH of SWF was then assessed. SWF with different pH (pH 4, 6, and 8) was incubated with various wound dressings for 24 h and the pH values of wound dressing contacting SWF were then determined using a pH meter. The pH after incubation in SWF with pH 4, 6, and 8 are shown in Figure 4A–C respectively. Unsurprisingly, we found that the wound dressing exposure had a minimal effect (<0.5 pH change) on SWF. For all the dressings, the pH change was within 0.5 units, except in the case of one alginate dressing (PRISMA), where a change of up to 1 unit was observed at pH of 4 and 6. The minimum volume of SWF that could be detected accurately without interference from each dressing was between 4 and 10 μL for all the dressings, except as follows: Aquacel Ag and Aquacel extra (170 μL); Hydrofera Blue (40 μL), PROMOGRAN PRISMA (6 mL); Iodoflex (5 mL); Medihoney (2.5 mL); Enluxtra (1.3 mL). It must also be noted that SWF is formulated in Tris buffer to mimic a buffer of carbonic acid and bicarbonate anion in human blood. It is possible that both buffer solutions diminish the effect of dressing in SWF pH.

### Use of DETEC^®^ pH to assess wound alkalinity

We tested a number of discarded wound dressings from chronic wound patients suffering from diabetic ulcers, pressure ulcers and venous ulcers using DETEC^®^ pH.

**Figure 5.**
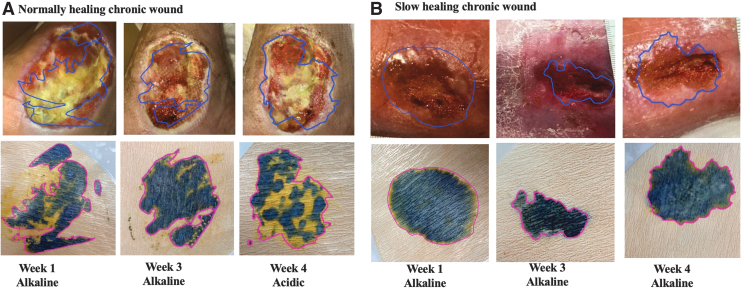
Clinical testing of DETEC^®^ pH in **(A)** normally healing wound and **(B)** slow healing chronic wound. The areas of pH reading are traced with *blue* and *red lines* on corresponding wound image and DETEC^®^ pH, respectively.

We found that DETEC^®^ pH can be used to assess wound pH distribution as shown in Figure 5. Interestingly, our device detected several acidic wounds which, at least partially, were covered with granulation tissue (Fig. 5A). Interestingly, the areas of granulation tissue coincided with acidic yellow pattern. On the other hand, our device detected many alkaline wounds, which typically lack granulation tissue (Fig. 5B). We found that the best representation of the wound condition was when the colors were read on the main body of the image excluding the edges. The presence of yellow and/or light green indicated an acidic wound. Absence of yellow and/or light green indicated an alkaline wound.

### The effect of wound alkalinity on wound size reduction rates

**Figure 6.**
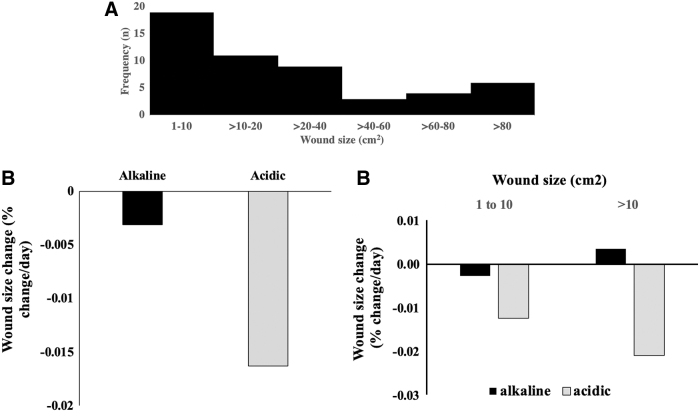
Effect of wound alkalinity on rate of change in wound size. **(A)** The distribution of wound sizes. **(B)** Comparison of rate of change in wound size between alkaline and acidic wounds was calculated as (wound size at visit 2 − wound size at visit 1)/(wound size at visit 1). **(C)** Influence of wound sizes on wound alkalinity-dependent rate of change in wound size.

**Figure 7.**
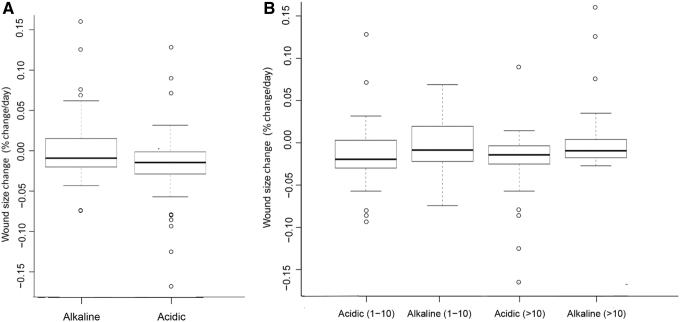
Box plot showing the distribution of wound size reduction rates based on wound alkalinity. **(A)** Distribution of wound size reduction rates between alkaline and acidic wounds. **(B)** Influence of wound sizes on the distribution of wound size reduction rates between alkaline and acidic wounds.

We first determined the distribution of patient numbers across various wound sizes (Fig. 6A). To determine whether pH mapping can be used as a prognostic indicator of wound healing activities, we compared the wound closure rates between alkaline wounds and acidic wounds. We found that upon analyzing all the wounds regardless of their individual sizes, the wound size reduction rates in acidic wounds were significantly larger than those in alkaline wounds with a *p*-value of 0.0069 (Fig. 6B; see also Fig. 7A). To carry out the test for significance, we used a two-sample nonparametric Wilcoxon rank sum test since the normality assumptions for two-sample t-test were violated for both alkaline and acidic wounds. Further categorization of the wounds based on their size showed that in each size range, acidic wounds had a greater size reduction rates than alkaline wounds at 10% level of significance (*p*-value of 0.0936 and 0.0233 for wound size 1–10 and >10 cm^2^, respectively). Note that this difference was more significant as the wound size increased (Fig. 6C; also see Fig. 7B). It must be noted that in both Figure 6B and C, the wound size during visit 2 was compared with that during the earlier visit. Hence, in cases where the wound was healing normally, this change was negative as the wound size was decreasing. Except, in the case of alkaline wounds that were >10 cm^2^, where this change was positive (Fig. 6C).

## Discussion

There is a critical need for rapid, easy-to-use, inexpensive wound assessment technologies that can accurately indicate the healing state of the wound. Although this information could be vital for a wound care specialist in selecting a suitable treatment, a device that could provide this information is, unfortunately, unavailable in the United States. Technologies that do exist in the realm of wound diagnostics rely on swabbing the wound, take at least 10 min to provide a reading, and could interfere with the normal flow of procedures in a busy wound clinic. Current clinical standard for wound assessment involves observation of clinical signs and symptoms, such as pain, erythema, edema, and purulence, as well as visual observations of the wound often followed by microbiological analysis of wounds.^51^ In the light of the fact that a biophysical parameter like wound pH is a very important indicator of the state of the wound environment, through this work we have presented an innovative approach of measuring wound environment by detecting wound alkalinity. Unlike previous approaches that directly tested the wound environment, we have documented the ability to measure and create a visual map of the pH environment on the wound contacting side of discarded wound dressings to identify the state of healing and a window into the short-term healing outcome based on the alkalinity level.^52–54^ This approach offers a number of advantages. First, it is noninvasive and does not involve any direct contact with the patient. Second, it does not interfere with any other procedure or treatment as the test can be done away from the patient. Third, avoiding contact with patients eliminates the risk of contamination of the patient's wound. In most of the cases, gently pressing our device against dressings is sufficient to obtain the reading. Lastly, all the above reasons contribute to minimizing patient discomfort while providing the clinician with a tool to make a prognosis on wound healing. Additionally, testing the dressing on the device should not interfere with regular clinical procedures as it can be done away from the patient following routine procedures stipulated for handling biological hazards and waste. However, it must be mentioned that the device provides a reverse map that may not pinpoint the specific location of acidity or alkalinity.

Our findings assume significance in the light of the fact that reliable, rapid, easy-to-use and yet inexpensive wound prognostics have not seen major developments over the years. However, it must be mentioned that wound treatments have seen a lot of progress, including in the therapeutic application of pH-altering strategies. A number of chronic wound treatments are centered on adjusting moisture, bacterial count, and altering the pH by reducing the alkalinity of the wound environment.^14,55,56^ Treatments like medicinal ointments, wound dressings, and debridement that involves removal of necrotic tissue to expedite healing also relies on alteration of wound pH to a less alkaline and more acidic level. A number of wound dressings are known to alter wound pH in this manner as shown in an earlier study.^45^ Some wound dressings contain acids, such as citric and acetic,^56^ while Manuka honey dressings are naturally low in pH and contain glauconic acid which accelerate healing by acidifying the environment.^14^ Honey is known to have antibacterial and anti-inflammatory properties in addition to its osmotic effect. These developments underscore the importance of pH in the overall process of wound treatment. Our results from *in vitro* testing support that the pH of the wound bed and the wound exudate that is soaked on to the dressing are comparable implying that we could possibly detect this pH to get information on the state of the wound. We also believe that the volume of SWF or wound exudate could depend on the dressing material and thickness. For example, Enluxtra™ is made of highly absorbent material with thickness of 0.5cm, requiring high volume of SWF to be able to use our device. On the other hand, Sorbact is thin and hydrophobic and only needed around 10uL of SWF to be detectable by our device. Interestingly, many dressing types tested in this study had insignificant effect on the output of DETEC^®^ pH suggesting that the device can be used on various types of dressings. However, it must be noted that PRISMA dressing that is designed to lower the wound pH to promote wound healing, had the highest effect on pH thus requiring the largest volume to overcome the interference.

From a prognostic standpoint, attempts have also been made to incorporate pH sensors into wound dressings. For example, dressing technologies have been developed to include pH indicators in them.^52–54^ However, the change in color on these dressings as the pH changes is irreversible making the pH indication aspect of the dressings irrelevant. Additionally, none of these developments has led to commercialization, except a skin pH meter which is used in clinical practice. Also, these wound contacting diagnostics could raise concerns of interference with the wound environment.

The value of pH as a biomarker has been harnessed for the detection of various conditions like urinary tract infections and vaginal infections.^30–33^ Its relevance in wound diagnostics has also been greatly emphasized in the past.^36,57^ However, the wound is a complex, nonhomogenous environment and most of these products can only provide the value of pH at any given point instead of the entire wound landscape.^35–37^ Also, previous studies have shown that when healing begins, wound environment typically changes from alkaline to neutral and then to an acidic state.^11,18,26,58^ While wound alkalinity is an important prognostic factor of nonhealing wounds, no major attempt has been made to link this to wound healing activities. While a number of colorimetric pH strips are commercially available, none of them can map out the pH on the test specimen. To the best of our knowledge, there is no publication on the use of pH strips for assessment of wound infection or healing. This could be due to the fact that wound fluids may contain blood clot, tissue debris, pus, etc., which may affect the color of pH strips. We, therefore, developed DETEC^®^ pH as an inexpensive card–like device with a test area that can contact a discarded wound contacting dressing and present a map of the alkalinity of the wound on the indicator area. In a clinical setting, this device could distinctly indicate acidity or alkalinity.^59^ Although cessation of inflammatory responses can be part of normal wound healing responses before wound maturation and epithelialization, an early onset of inflammation cessation, at least partially, contributes to nonhealing wounds. Chronic wounds exhibit a pH around 7.15–8.90 at the wound bed, creating a slightly alkaline environment.^12^ Metalloproteinases degrade proteins more rapidly in basic conditions, consuming more oxygen from the tissue to speed up the process.^2,29^ Therefore, it is clinically favorable to have a more acidic environment to slow metalloproteinase degradation rates, decrease abnormal collagen in the wound bed, increase fibroblast activity, and enhance the toxicity of the environment to bacteria for effective wound treatments. Our observation of acidic patterns in wounds with faster healing rate than those in alkaline has been corroborated by previous findings.^17,22,25,28,35,57^ We found that acidity detected by DETEC^®^ pH was indicative of wounds that exhibited a greater reduction in wound size over a short-term (2-week) period regardless of the initial size of the wound. This suggests that the device can be used to prognosticate wound healing rate, at least over a short term and potentially help doctors determine if the patient is on the right treatment course. While standard linear wound measurements could suffer from variability between readings, we have used a large number of patients to show the influence of wound alkalinity on wound size reduction rates. Additionally, future studies could use a more reproducible analytic device that would provide better wound size measurement and overall analyses.

To the best of our knowledge, DETEC^®^ pH is the first device designed to rapidly determine wound alkalinity without direct patient contact. Furthermore, our results support that DETEC^®^ pH, although not a stand-alone device and cannot be used on dry dressing, can be used as a prognostic tool that can screen chronic wounds and predict short-term wound healing activities and thus their reduction rates. Although, we do not know the direct impact of biofilms or presence of bacteria on device measurement, as wound exudate is a buffered solution, it is expected to be limited. Future work with this device will entail determination of this and long-term healing outcome based on the device output with larger patient numbers. Additionally, we will explore a quantitative analysis of pH ratios along with studying the effect of various interferents in the future. Our studies have shown that very small amounts (5 μL) of wound exudate are sufficient to produce reliable readings. Larger quantities could improve the reliability and will be considered in future studies with more patient samples. This device could potentially impact clinical wound care by providing an early indication of wound healing status for wound care providers to determine whether treatment is effective. Such information can be vital in aiding the clinician make informed treatment decisions that could significantly reduce repeated hospital visits and treatment costs. Finally, DETEC^®^ pH can be used as an aid for home health care nurses or health care providers who take care of patients with chronic wounds outside clinical facilities.

## Innovation

Acidic and alkaline wound environments indicate active healing and cessation of healing responses, respectively. While steering wounds away from alkaline environment is a common strategy for accelerating wound healing responses, there is no commercial available product that can map the nonhomogeneous pH environment across the wounds and then prognosticate the wound healing activities. DETEC^®^ pH fills this gap, by avoiding patient contact and testing discarded wound dressings to generate a map of the alkalinity levels that can prognosticate short-term wound healing rates and screen nonhealing wounds. This device can serve as a visual aid for wound care specialists to forecast wound healing activities and make informed treatment decisions in the wound care process.

**Key Findings**

- DETEC^®^ pH can map the alkalinity of the wound exudate adsorbed onto discarded wound dressings without direct patient contact.
- This color map can be used to assess the complex wound environment in chronic wounds as alkaline or acidic wounds.
- Alkaline wounds exhibited slower short-term healing rate than acidic wounds as evidenced by wound-size reduction.
- The device is a rapid point-of-care wound healing prognostic and may be used as an aid to screen nonhealing wounds outside clinics.

## About the Authors

**Hong Vu, MS** is a scientist at Progenitec Inc. Her expertise is in biochemistry and analytical chemistry with focus on medical device development. **Ashwin Nair, PhD** is a scientist and project leader at Progenitec Inc., with expertise in foreign body reactions to materials and tissue regeneration techniques. **Wenjing Hu, PhD** is a scientist and CEO of Progenitec Inc. Her expertise is in medical devices and body's response to implanted biomaterials. **Lan Tran, BS** is a technician at Progenitec Inc who executed a number of analytical studies in this project. Her undergraduate training is in biological chemistry. **Suvra Pal, PhD** is an Assistant Professor in The Department of Mathematics at the University of Texas at Arlington with expertise in the area of biostatistics and predictive modeling. **Jon Senkowsky, MD** is a vascular surgeon with over 30 years of clinical experience in wound management. He provides care for chronic and slow healing wound patients suffering from vascular disease, venous stasis and diabetes using many techniques and products. **Liping Tang, PhD** is a bioengineering professor at the University of Texas at Arlington. His expertise covers a broad area of biocompatibility, biomaterials, tissue engineering, drug delivery, inflammation imaging, infection and stem cell therapies.

## Abbreviation and Acronym

SWF: simulated wound fluid
